# Plasma MicroRNA Signature Panel Predicts the Immune Response After Antiretroviral Therapy in HIV-Infected Patients

**DOI:** 10.3389/fimmu.2021.753044

**Published:** 2021-11-23

**Authors:** Jun-Nan Lv, Jia-Qi Li, Ying-Bin Cui, Yuan-Yuan Ren, Ya-Jing Fu, Yong-Jun Jiang, Hong Shang, Zi-Ning Zhang

**Affiliations:** ^1^ National Health Commission (NHC) Key Laboratory of AIDS Immunology (China Medical University), National Clinical Research Center for Laboratory Medicine, The First Affiliated Hospital of China Medical University, Shenyang, China; ^2^ Key Laboratory of AIDS Immunology, Chinese Academy of Medical Sciences, Shenyang, China; ^3^ R&D Department, Beijing Quantobio Star Biotechnology Co., Ltd., Beijing, China

**Keywords:** HIV, immunological non-responders, microRNA, biomarker, ART

## Abstract

**Background:**

Approximately 10–40% of people with human immunodeficiency virus (HIV) infection are unable to obtain successful improvements in immune function after antiretroviral therapy (ART). These patients are at greater risk of developing non-acquired immunodeficiency syndrome (AIDS)-related conditions, with the accompanying increased morbidity and mortality. Discovering predictive biomarkers can help to identify patients with a poor immune response earlier and provide new insights into the mechanisms of this condition.

**Methods:**

A total of 307 people with HIV were enrolled, including 110 immune non-responders (INRs) and 197 immune responders (IRs). Plasma samples were taken before ART, and quantities of plasma microRNAs (miRNAs) were determined using reverse transcriptase-quantitative polymerase chain reaction (RT-qPCR). Candidate biomarkers were established through four phases: discovery, training, validation, and blinded test. Binary logistic regression was used to analyze the combined predictive capacity of the identified miRNAs. The effect of one miRNA, miR-16-5p, on T cell function was assessed *in vitro*.

**Results:**

Expression of five miRNAs (miR-580, miR-627, miR-138-5p, miR-16-5p, and miR-323-3p) was upregulated in the plasma of INRs compared with that in IRs. Expression of these miRNAs was negatively correlated with both CD4^+^ T cell counts and the increase in the proportion of CD4^+^ T cells after one year of ART. These five miRNAs were combined in a predictive model, which could effectively identify INRs or IRs. Furthermore, we found that miR-16-5p inhibits CD4^+^ T cell proliferation by regulating calcium flux.

**Conclusion:**

We established a five-miRNA panel in plasma that accurately predicts poor immune response after ART, which could inform strategies to reduce the incidence of this phenomenon and improve the clinical management of these patients.

## Introduction

Currently, most people infected with the human immuno-deficiency virus (HIV) who receive antiretroviral therapy (ART) respond well to treatment, and are able to maintain an undetectable viral load (1). However, approximately 10–40% of people with HIV are unable to obtain improvements in immune function (also called immune reconstitution) even when the viral load is under control after ART. These people are referred to as immune non-responders (INRs) in a clinical setting (2–4). Compared with immune responders (IRs), in whom both CD4^+^ T cell counts and function are restored, INRs remain at greater risk of developing non-acquired immunodeficiency syndrome (AIDS)-related-events, with the accompanying increased morbidity and mortality (5–7). There is a pressing need to identify the underlying mechanisms and predictive factors of poor immune responses to ART, which could reduce the incidence of this phenomenon and improve the clinical management of INRs.

Previous research has shown that immunological, genetic, and viral factors are related to a poor immune response to ART, such as defective bone marrow and thymus functions, chronic immune activation and inflammation, polymorphisms in the CD14 and toll-like receptor genes, and accumulation of lipid metabolites in plasma (8–10). Some clinical indicators, such as age (11), baseline or nadir CD4^+^ T cell counts (12, 13), activated programmed cell death protein 1 (PD-1)^+^ CD4^+^ T cells (14), metabolomic signatures (15), and gene polymorphisms (16), are predictors for recovery of CD4^+^ T cells after ART. However, the application of these indicators to predict the prognosis of patients with comparable age and baseline CD4^+^ T cells has limitations. Various strategies have been attempted to improve the level of immune reconstitution in INRs (4). In the current study, we aimed to develop a practical and effective predictor of immune responses in people with HIV receiving ART.

MicroRNAs (miRNAs) are small, endogenous RNAs that regulate gene expression at the post-transcriptional level. Studies have shown that endogenous circulating miRNAs are stable blood-based biomarkers because of their chemical stability and resistance to RNase activity (17–19). Different mechanisms have been identified to protect circulating miRNAs from degradation. First, miRNAs form complexes with proteins such as argonaute-2 or nucleophosmin-1 (20, 21); Second, miRNAs are packaged inside small vesicles that are derived from endosomal membrane compartments or shed directly from the plasma membrane (22); Third, miRNA modifications make them resistant to RNase activity (23). Several studies have indicated that these circulating miRNAs are promising biomarkers for the prognosis of a variety of diseases, including liver, renal, cardiovascular, infectious diseases, and different tumor types (24–29). In HIV infection, circulating miRNAs in plasma or serum could potentially be used as blood-based biomarkers for the detection of early infection with HIV-1 (30), HIV disease progression (31–33), HIV-associated nervous system disorders (34–36), and liver injury in people with HIV (37, 38). As next-generation, non-invasive clinical biomarkers (39, 40), circulating miRNAs are worth investigating for their potential use in developing biomarkers and better understanding the underlying mechanisms governing the immune response to ART. Recently, Fu and colleagues compared miRNAs in the plasma samples of 13 IRs and 11 INRs. They identified let-7d-5p as a potential miRNA biomarker for non-response to therapy (41). However, studies with larger cohorts of patients are needed to establish promising miRNA biomarkers that can be used to predict INRs reliably, and to elucidate the underlying molecular mechanisms of immune reconstitution failure.

In our study, baseline (pre-ART) plasma miRNA profiles were compared between 110 INRs and 197 IRs. To our knowledge, this is the first large-scale study investigating miRNAs as potential predictive biomarkers of response to ART. Our study also provides new insights into the underlying mechanisms of immune recovery after therapy for HIV infection.

## Methods

### Study Population and Experimental Design

**Figure 1:**
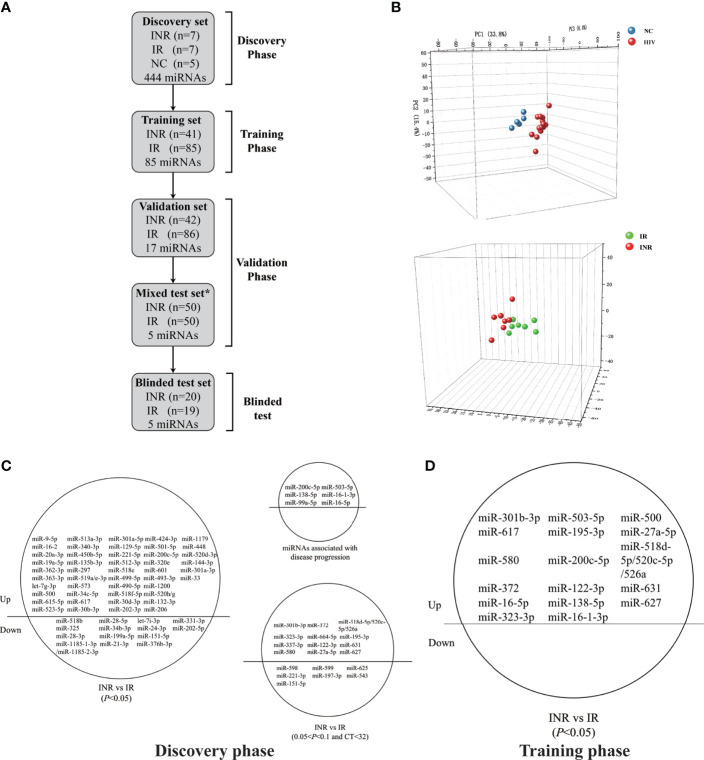
Circulating microRNA (miRNA) levels can predict differences in the extent of immune reconstruction after antiretroviral therapy (ART) in people with human immunodeficiency virus (HIV) infection. **(A)** Diagram of sample collection and miRNA screening in the four phases of our study. Plasma miRNA expressions of immune non-responders (INRs, n = 110), immune responders (IRs, n = 197), and HIV-negative controls (NCs, n = 5) were detected in the discovery, training, validation, and blinded test phases using fluorescent reverse transcriptase- quantitative polymerase chain reaction (RT-qPCR). *Samples in the mixed test set were obtained from the training and validation set. **(B)** Principal components analysis (PCA) of 60 differentially-expressed miRNAs from INRs, IRs, and NCs in the discovery phase. **(C)** The 60 differentially-expressed miRNAs (*p* < 0.05), 19 miRNAs (0.05 < *p* < 0.1, CT < 32) and six reported miRNAs chosen for evaluation in the training phase. **(D)** The 17 miRNAs significant differential expressed (*p* < 0.05) between IRs and INRs from the training phase.

Overall, 307 HIV-infected patients were recruited from a cohort of HIV-infected patients who were followed up at an AIDS Clinic. The following criteria were employed for study inclusion: (i) receiving ART for more than one year; (ii) baseline CD4^+^ T cell counts between 200 and 500 cells/μL before ART; (iii) aged between 18 and 60 years; (iv) had no coinfection with HBV or HCV. There are differences in miRNA expression and the immune response in chronic/long-term HIV-1 infection *vs*. early/recent HIV-1 infection. Hence, patients who were treated during early HIV infection were excluded. The 307 patients were recruited for the discovery, training, validation, and blinded test phases of our study to identify miRNA biomarkers (**Table 1** and **Figure 1A**). Based on the CD4^+^ T cell increase in percentages after one year of therapy from baseline, patients were classified as INRs (increase in CD4^+^ T cells < 20% over baseline levels after one year of ART, n = 110) (42–44) and IRs (increase in CD4^+^ T cells > 30% over baseline levels after one year of ART, n = 197) (45, 46). The demographic and clinical characteristics of individuals in each group were recorded. There were no substantial differences in sex, age, ethnicity, viral subtype, baseline CD4^+^ T cell counts, or viral load between INRs and IRs. In addition to the study population with HIV, five sex- and age-matched HIV-negative control individuals (NCs) were enrolled in the discovery phase. Baseline plasma samples were taken from all individuals included in our study. In those with HIV, these samples were taken before ART, and the median (interquartile range, IQR) time before ART was 9 (6–18) days. The studies involving human participants were reviewed and approved by the ethical review committee from The First Hospital of China Medical University, Shenyang, China. Written informed consent to participate in the study was obtained from all individuals.

**Table 1:** Demographic and clinical characteristics of patients included in this study.

Group	Characteristic	Discovery phase	*p*-value	Training phase	*p*-value	Validation phase	*p*-value	Blinded phase	*p-*value
INRs	n	7		41		42		20	
	Male, n (%)	7(100)	>0.999	41 (100)	0.550	39 (93)	0.973	20 (100)	0.106
	Age, median (IQR)	27(25–38)	0.269	32 (26–39)	0.845	30 (25–41)	0.632	29 (26–40)	0.180
	Han ethnic, n (%)	6 (86)	0.515	36 (88)	0.478	37 (88)	0.337	17 (85)	0.316
	Subtype CRF01AE, n (%)	6 (86)	>0.999	27 (66)	0.448	26 (62)	0.976	13 (65)	0.333
	Baseline CD4 (cells/μL), median (IQR)	316 (295–447)	0.068	329 (282–409)	0.235	330 (271–403)	0.302	297 (245–375)	0.682
	ART CD4 (cells/μL), median (IQR)	341 (293–402)	0.004	336 (282–402)	<0.001	335 (261–392)	<0.001	324 (256–384)	<0.001
	Viral load (log copies/mL), median (IQR)	4.57 (4.39–4.77)	0.383	4.37 (3.92–4.67)	0.081	4.24 (3.74–4.53)	0.067	4.23 (3.53–4.66)	0.424
IRs	n	7		85		86		19	
	Male, n (%)	7 (100)		82 (96)		80 (93)		16 (84)	
	Age, median (IQR)	35 (29–41)		31 (27–40)		30 (25–42)		32 (27–49)	
	Han ethnicity, n (%)	5 (71)		78 (92)		70 (81)		18 (95)	
	Subtype CRF01AE, n (%)	6 (86)		50 (59)		53 (62)		15 (79)	
	Baseline CD4 (cells/μL), median (IQR)	249 (217–397)		310 (266–350)		315 (279–350)		303 (269–343)	
	ART CD4 (cells/μL), median (IQR)	590 (476–815)		526 (450–639)		574 (476–629)		509 (414–675)	
	Viral load (log copies/mL), median (IQR)	4.38 (3.98–4.94)		4.55 (4.11–4.93)		4.47 (3.89–4.77)		4.36 (3.98–4.77)	

ART, antiretroviral therapy; INRs, immunological non-responders; IRs, immunological responders; IQR, interquartile range; n, number. p-value: INRs compared with IRs.

### Plasma miRNA Quantification

Plasma samples were collected by centrifuging fresh blood at 2000×*g* for 10 min and were isolated and stored at -80°C. Quantities of miRNA were determined using SYBR dye-based reverse transcriptase-quantitative polymerase chain reaction (RT-qPCR; QuantoBio, Beijing, China). Briefly, synthetic *Caenorhabditis elegans* miRNA (cel-miR-67-3p) was added to each plasma sample as an exogenous control (EC) before RNA extraction (24) using a whole-miRNA isolation kit according to manufacturer’s instructions (BioChain, Newark, CA, USA). In order to quantify miRNAs, *Escherichia coli* poly(A) polymerase and oligo (dT) were first used to synthesize complementary DNA (cDNA). High-throughput miRNA profiling was undertaken using TB Green Premix Ex Taq II (TaKaRa Biotechnology, Shiga, Japan) with miRNA-specific primers and universal primers (UPM). The EC and UPM sequences are listed in **Supplemental Table 1**. Data analysis was carried out using OmicsOffice^®^, which is an extension to TIBCO Spotfire^®^ (Palo Alto) (47).

### Transfection, Transcriptome Sequencing and Proteomic Analysis

To investigate the functions of miR-16-5p, CD3^+^ T cells were negatively isolated (StemCell Technologies, Vancouver, Canada) from the peripheral blood mononuclear cells of HIV-infected patients. The miR-16-5p mimic and mimic control (GenePharma, Shanghai, China) were transfected into separate CD3^+^ T cells respectively at a final concentration of 10 nM with Lipofectamine RNAiMAX Transfection Reagent (Thermo Fisher Scientific, Waltham, MA, USA) according to the manufacturer’s protocol. The sequences of the miR-16-5p mimic and control are listed in **Supplemental Table 1**. For transfection efficiency detection, total miRNAs were extracted using the RNeasy Plus Micro Kit (Qiagen, Stanford, VA, USA) after transfection for 40 h and were reverse-transcribed using Mir-X miRNA First-Strand Synthesis Kit (TaKaRa Biotechnology). Expression levels of miRNAs were evaluated using TB Green Premix Ex Taq II (TaKaRa Biotechnology). To identify miR-16-5p target genes, transcriptome sequencing of miR-16-5p mimics and mimic control in CD3^+^ T cells was performed after transfection for 40 h. A transcriptome sequencing service was provided by OE Biotech Co. Ltd. (Shanghai, China). Libraries were constructed and sequenced on the Illumina HiSeq X Ten platform (Illumina, San Diego, CA, USA). To identify miR-16-5p target genes at the proteomic level, the miR-16-5p mimic and mimic control were respectively transfected into the Jurkat cell line. Liquid chromatography-tandem mass spectrometry was carried out as described in detail previously (PTM Biolab, Hangzhou, China) (48).

### Flow Cytometry

After transfection for 40 h, primary CD3^+^ T cells were stimulated using Dynabeads™ Human T Activator CD3/CD28 (bead to cell ratio, 1:4; Thermo Fisher). Proliferation and apoptosis were carried out according to previously described methods (49). In order to detect calcium (Ca^2+^) flux, CD3^+^ T cells were stained with LIVE/DEAD™ dye (Thermo Fisher), followed by loading with Fluo-4-AM, Fura-red, and Pluronic F-127 (Thermo Fisher) for 30 min. Cells were then labeled with antibodies against CD3, CD4, and CD8 for 20 min. To record Ca^2+^ flux, a baseline fluorescence signal was acquired for 90 s. Following this period, the cells were stimulated with soluble anti-CD3/CD28 (10 μg/mL) (50), after which fluorescence signal recording was continued for 300 s. Ionomycin (1 μg/mL) was used to elicit a maximum response for another 120 s. For quantification of Ca^2+^ flux, the ratio of Fluo-4 or Fura-red at the peak of the response relative to the baseline was determined (51). Cells were detected using the LSR II flow cytometer (BD Biosciences, San Jose, CA, USA) and data were analyzed using FlowJo software (Ashland, OR, USA).

### Statistical Analysis

GraphPad Prism 8 (GraphPad, San Diego, CA, USA) and SPSS 21.0 (IBM, Armonk, NY, USA) were used to conduct statistical analysis and generate graphs. The Mann-Whitney U-test was used to compare age, CD4^+^ T cell counts, and viral loads between INRs and IRs. The comparisons of sex, ethnicity, and viral subtype between INRs and IRs were conducted using Fisher’s exact test or Chi-square test.

Pearson correlation analysis was performed to evaluate the correlation between miRNA expression and CD4^+^ T cell counts. Principal components analysis (PCA) was used to analyze the distribution of miRNAs between groups using Origin 9.1 software (OriginLab Corporation, Northampton, MA, USA). Receiver-operating characteristic (ROC) analysis was used to evaluate the performance of miRNA biomarkers as predictors of immune reconstitution. The predictive value was expressed as the area under the curve (AUC). Binary logistic regression was used to assess the predictive capacity of the combined miRNA model, constructed using the following formula: Logit (P) = β_0_+β_1_*Ct_1_+β_2_*Ct_2_+…+β_n_*Ct_n_ (where β is the partial regression coefficient, and Ct is the delta cycle threshold (CT) of each miRNA), with the probability of success expressed as: y = 1/(1+e^-logit(P)^) (52). Patients were sorted into two groups using the optimal cut-off value, determined according to the maximum Youden Index. The maximum Youden Index is defined as J = max_t_{sensitive(t)+specificity(t)-1}, where t is the maximum classification threshold of J (53). For *in vitro* experiments, paired t-tests were used to assess the differences in T cell function and Ca^2+^ ratio between miR-16-5p mimic and control T cells. A *p-*value of < 0.05 was considered statistically significant.

## Results

### Plasma miRNAs Profile Discriminating INRs and IRs

To determine specific miRNAs with predictive capacity for the response to ART, a total of 307 people with HIV infection on ART (including 110 INRs and 197 IRs) were enrolled, and sorted into groups corresponding to the four phases of our study. In the first phase (discovery), 444 miRNAs were tested among seven INRs, seven IRs, and five NCs. According to the PCA analysis, people with HIV infection who received ART (INRs and IRs) and NCs could be distributed into two distinct clusters (**Figure 1B**), which are consistent with results from the study by Fu and colleagues (41). Notably, the miRNA profile could be used to differentiate INRs and IRs by PCA analysis despite no difference in baseline CD4^+^ T cells between INRs and IRs (**Figure 1B**).

During the discovery phase, 60 miRNAs were found to show differential expression between INRs and IRs (*p* < 0.05, fold change > 1.5). Among them, the expression of 46 miRNAs was upregulated in INRs, and the expression of remaining 14 miRNAs was downregulated compared with those in IRs (**Figure 1C**). The expression profiles of the 60 miRNAs, 19 more miRNAs with *p* < 0.1 and CT < 32, and six miRNAs found previously to be associated with HIV disease progression (54–56) were tested in the second phase (an independent training cohort). In this training phase, expression levels of all 85 miRNAs were assessed in 41 INRs and 85 IRs. After the training phase, the expression of 17 miRNAs was found to be significantly higher in INRs than in IRs (all *p* < 0.05, **Figure 1D**).

### Validation of Five Differentially Expressed miRNAs Between INRs and IRs

**Figure 2:**
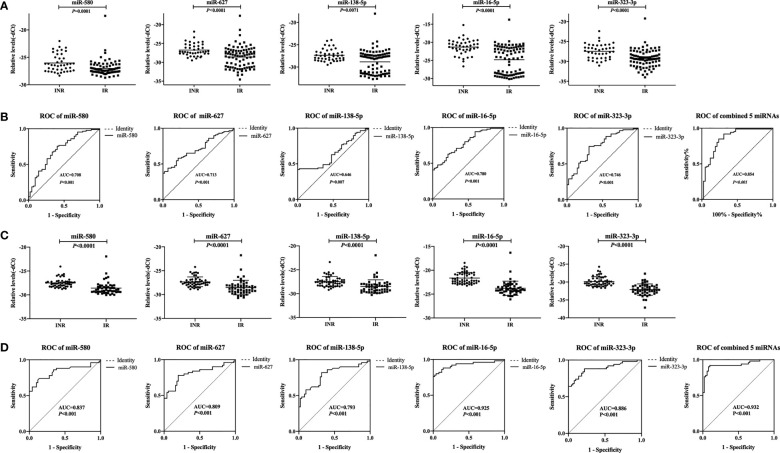
Relative expression levels of five plasma microRNAs (miRNAs) and analyses of receiver operating characteristic (ROC) curves in the validation phase. **(A)** Relative expression of five miRNAs in immune non-responders (INRs, n = 42) and immune responders (IRs, n = 86) in the validation set. **(B)** Analyses of ROC curves of five miRNAs and our combined five-miRNA panel performed in the validation set. **(C)** Relative expression levels of five miRNAs in INRs (n=50) and IRs (n=50) in the mixed test set. **(D)** Analyses of ROC curves of five miRNAs and our combined five-miRNA panel as performed in the mixed test set. The area under the ROC curve (AUC) value represents the diagnostic capability.

In the third phase (validation), the expression of the 17 miRNAs identified during the training phase was measured in a cohort of 42 INRs and 86 IRs. Among them, five miRNAs (miR-580, miR-627, miR-138-5p, miR-16-5p, and miR-323-3p) with increased expression in the training phase (**Supplementary Figure 1**) were found to be significantly upregulated in INRs compared with IRs (*p* < 0.01, fold change > 1.5, CT value < 35, **Figure 2A**).

Thus, we defined these miRNAs as potential biomarkers that be able to predict the immune response after ART. Analyses of ROC curves demonstrated that the AUC of each of these five miRNAs to be > 0.5, and the AUC of all five miRNAs combined exhibited a predictive value up to 0.854 (95% confidence intervals [CI], 0.778–0.930; *p* < 0.001; **Figure 2B**).

To assess if batch processing might have affected our results, the training set and validation set were combined. For each sample, there is a detection ID number (i.e., IR 1), which could be linked to the patient ID number (i.e., 300504). The samples with the top 25 detection ID numbers from IRs and INRs in the training set and the validation set were included, which added up to 50 IRs and 50 INRs. These 100 patients formed a mixed test set to detect the expression of the five miRNAs. The differential expression of these miRNAs between INRs and IRs, as established through the first three phases, was confirmed (**Figure 2C**). Analyses of ROC curves showed that these five miRNAs could be used to differentiate INRs from IRs (AUC = 0.932; 95% CI, 0.878–0.986; **Figure 2D**).

### Correlation of miRNA Expression With CD4^+^ T Cells in People With HIV

**Figure 3:**
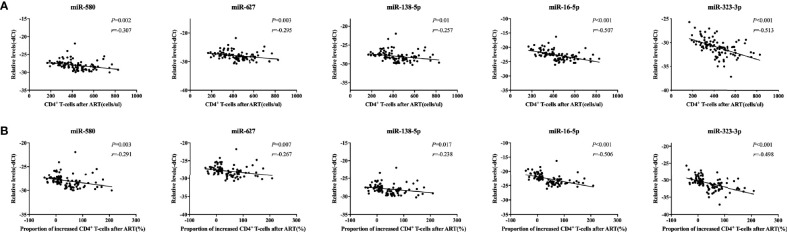
Correlations between relative expression of five microRNAs (miRNAs) and CD4^+^ T cell counts in people with human immunodeficiency virus (HIV) infection. Relative expression of all miRNAs was significantly negatively correlated with the **(A)** the number of CD4^+^ T cells and **(B)** the proportion of CD4^+^ T cells after antiretroviral therapy (ART) in immune non-responders (INRs, n = 50) and immune responders (IRs, n = 50) in the mixed test set (*p* < 0.05). The increase in the proportion of CD4^+^ T cells was calculated using the following formula: (CD4^+^ T cell count after ART – CD4^+^ T cell count at baseline)/CD4^+^ T cell count at baseline. The y-axis represents the relative expression levels of each miRNA.

Next, we analyzed the correlation between the expression of each of the five miRNAs and CD4^+^ T cell counts or the increase in the proportion of CD4^+^ T cells after one year of ART in the same mixed test set for validation of the five miRNAs. Our results showed that the expression levels of the miRNAs were negatively correlated with both CD4^+^ T cell counts and the increase in the proportion of CD4^+^ T cells after one year of ART (*p* < 0.05, **Figures 3A, B**). These findings suggested that expression of the five miRNAs correlated with the recovery of CD4^+^ T cell counts after ART in people with HIV infection.

### A Validated Model to Predict INRs and IRs

**Figure 4:**
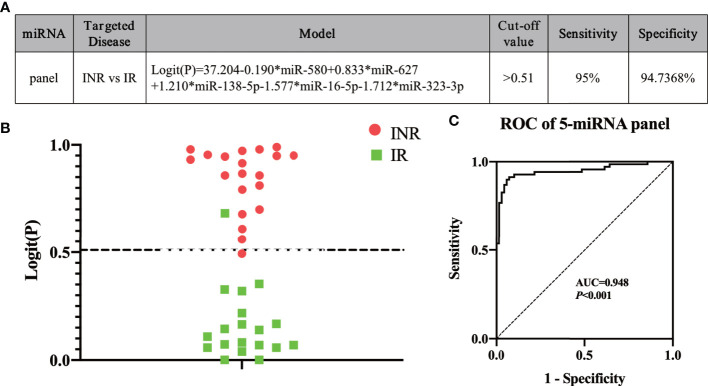
Using our five-miRNA panel to predict immune non-responders (INRs) and immune responders (IRs) in a blinded test. **(A)** The model and **(B)** the Logit(P) to identify INRs and IRs in blinded test sets (n = 49). The dashed line separates INRs and IRs by the cut-off value. **(C)** Analyses of receiver operating characteristic (ROC) curves of the five-miRNA panel, generated by sample analysis from the mixed and blinded test sets.

To construct an optimal predictive model, the five miRNAs (miR-580, miR-627, miR-138-5p, miR-16-5p, and miR-323-3p) were combined in a model to identify INRs or IRs based on the result of the mixed test phase (**Figure 4A**). The cut-off value was set as 0.51 according to the maximum Youden Index, which was calculated based on data from the mixed test set. Logit (P) was calculated using the expression of the five miRNAs based on the model (**Figure 4A**). If y, which equaled to 1/(1+e^-logit(p)^) (53), was more than 0.51, then a patient was identified as an INR. Otherwise, the patient was identified as an IR. To test the efficacy of the predictive model, a blinded test was conducted. miRNA expression in the plasma of 49 HIV-infected patients was measured before ART; 19 of 20 INRs and 18 of 19 IRs were predicted correctly at a sensitivity of 95.0% and specificity of 94.7% (**Figure 4B**). Finally, calculation of the data from the mixed test set and blinded test set produced an AUC value of 0.948 (95% CI, 0.909–0.988; sensitivity = 92.9%, specificity = 91.3%) based on our model (**Figure 4C**). These data indicated that our miRNA model could effectively predict INR or IR status before ART.

### miR-16-5p Suppresses T Cell Proliferation by Regulating Ca^2+^ Flux

Finally, we investigated whether these five ‘signature’ miRNAs could help to elucidate the potential mechanisms of different types of immune recovery. Among the five miRNAs, miR-16-5p was selected because: (i) it controlled most of the pathways that were jointly regulated by the five miRNAs (DINAN-microT-CDS/Tarbase, **Supplementary Figure 2**), (ii) miR-16-5p had the largest AUC value among the five miRNAs in analyses of ROC curves in the validation and mixed testing phases. Therefore, we used miR-16-5p as a test case to explore its role in immune reconstitution.

**Figure 5:**
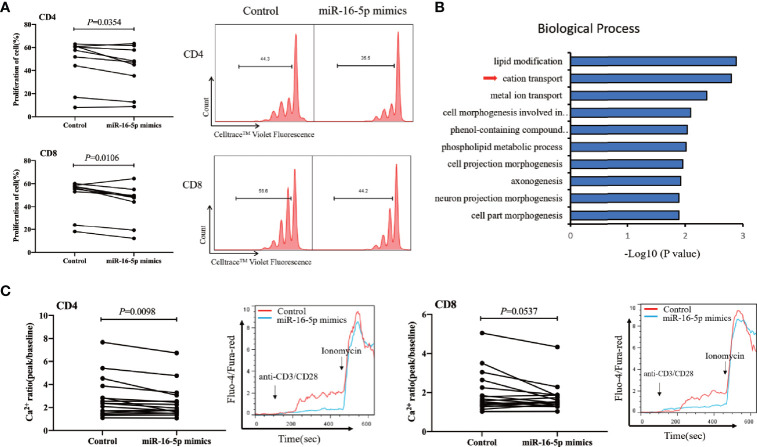
miR-16-5p overexpression suppresses T cell proliferation and Ca^2+^ flux. **(A)** T cells from people with human immunodeficiency virus (HIV) infection (n = 9) after antiretroviral therapy (ART) were transfected with miR-16-5p mimic or control. Representative histogram (right) showing proliferation of CD4^+^ T cell (top) and CD8^+^ T cell (bottom), measured using Cell Trace™ Violet. **(B)** Analysis of differentially expressed genes (using DAVID) between miR-16-5p mimic and control based on the transcriptome. **(C)** Ca^2+^ flux in CD4^+^ T cells (left) and CD8^+^ T cells (right) assessed using flow cytometry at baseline and then after the addition of anti-CD3 and anti-CD8 antibodies, presented as the ratio of fluorescence at the peak to fluorescence at baseline (n = 16).

We found that miR-16-5p overexpression in CD3^+^ T cells from people with HIV infection receiving ART (**Supplementary Figure 3**) was associated with significantly decreased proliferation of both CD4^+^ and CD8^+^ T cells compared with that in controls (*p* = 0.035 and *p* = 0.011 for CD4^+^ and CD8^+^ T cells, respectively) (**Figure 5A**). However, miR-16-5p overexpression did not appear to affect T cell apoptosis significantly (**Supplementary Figure 4**). Transcriptome analysis (n = 6) revealed that miR-16-5p to be involved in the cation transport and metal ion transport pathways (**Figure 5B**). *In vitro*, miR-16-5p overexpression diminished the magnitude of maximal Ca^2+^ peaks in CD4^+^ T cells (n = 16, *p* = 0.0098) but not in CD8^+^ T cells significantly (n = 16, *p* = 0.0537; **Figure 5C**). Analyses of the proteins with reduced expression in miR-16-5p overexpressed Jurkat cells revealed that proteins regulating T cells proliferation and Ca^2+^ influx were involved, such as matrix metalloproteinase-9 (MMP-9) (**Supplementary Figure 5**) (57). These results indicated that miR-16-5p suppressed the proliferation of CD4^+^ T cells, likely through regulating Ca^2+^ transport, which may affect immune recovery in patients on ART.

## Discussion

We discovered a robust set of miRNA biomarkers that can be used to predict the immune response. Recently, the clinical outcomes of individuals with HIV have improved owing to advances in ART (58). However, until now, poor immune reconstitution after ART in INRs has limited the improvements in outcomes (2, 59, 60). These predictive miRNA biomarkers may help to improve the early identification of INRs and develop clinical approaches to reduce morbidity and mortality in people with HIV infection.

Circulating miRNAs have essential roles in the diagnosis, prognosis, and evaluation of the therapeutic efficacy of different diseases (61, 62). During HIV-1 infection, the miRNA profile of the host changes, and miRNA expression varies with disease progression (40). To identify the specific circulating miRNAs in plasma that could be used to predict the immune responses after ART, we recruited a relatively large cohort of 307 HIV-infected patients (110 INRs and 197 IRs). Uniform definitions of IRs and INRs are lacking, and different definitions from the literature were summarized by Yang and colleagues recently (4). They stated that INRs could be defined by a failure to meet: (i) the prescribed CD4^+^ T cell counts (e.g., > 200, > 250, > 350, > 400, or > 500/µL); (ii) the prescribed increases in the percentage of CD4^+^ T cells over baseline (e.g., > 5%, > 20%, or > 30%); (iii) the prescribed increase in CD4^+^ T cell counts over baseline (e.g., > 50, > 100, or > 400/µL). In our study, the definition of IR and INR was set according to the increase in the percentage of CD4^+^ T cells over baseline. We used a robust study design with four phases in order to identify a five-miRNA panel that could predict the immune response reliably. During the blinded test phase, the diagnostic performance of our miRNA panel had better discriminatory power in predicting immune reconstitution after ART.

Plasma or serum miRNA expression profiles have been reported in hepatitis-B virus (HBV), hepatitis-C virus (HCV), and human T-cell leukemia virus type 1 (HTLV-1) (63–67). The five-miRNA panel as a biomarker found in our study has not been reported in other viral infections. Among the five plasma miRNAs in our panel (miR-580, miR-627, miR-138-5p, miR-16-5p, and miR-323-3p), serum miR-16 was higher in patients with chronic hepatitis C compared to healthy controls (68). None of the other four miRNAs expressed in plasma or serum have been reported to be altered in HBV, HCV, HTLV-1 infection. Because of the relatively low prevalence of HIV-2 infection, there were no reports regarding circulating plasma or serum miRNA profiles in HIV-2 infection to the best of our knowledge. These results suggested the specificity of the combined five-miRNA panel in predicting immune responses in people with HIV who received ART. Even though there were no significant differences in baseline CD4^+^ T cell counts and age between IRs and INRs, our five-miRNA panel could predict response to ART in patients effectively.

ART initiation during early HIV infection is associated with an enhanced likelihood of recovery of CD4^+^ T cell counts (69). Also, miRNA expression and the immune response in chronic HIV-1 infection *vs*. early HIV-1 infection are different (40). Hence, the proportion of early HIV infected patients in the cohort would influence research results. To avoid this problem, patients who were treated during early HIV infection (e.g., with documented or clearly remembered time of infection) were excluded. For most of the patients who were not able to recall the exact time of HIV infection, we postulated that the proportion of early infections was low because they had a long history of high-risk behavior. We did not observe alterations in the expression of previously reported miRNAs in patients who initiated ART in the acute phase of HIV infection (e.g., let-7d-5p) (41). These data may suggest that our miRNA biomarkers are suitable for most patients who receive ART during chronic HIV infection.

The underlying mechanisms for incomplete immune reconstitution have still not been clarified. Biomarker studies can improve clinical diagnosis and provide clues to the mechanism of immune recovery after ART. Among the five miRNAs in this panel, miR-627, miR-138-5p, miR-16-5p, and miR-323-3p have previously been identified as biomarkers in various immune and inflammatory responses (70–74). We selected miR-16-5p to study the possible mechanism because it had the best diagnostic performance in the validation set and mixed testing set, and it controlled most of the pathways jointly regulated by the five miRNAs in our biomarker panel. Further analyses showed that miR-16-5p could participate in the pathways closely related to the growth, proliferation, and apoptosis of cells. We found that miR-16-5p could suppress the proliferation of T cells, a pivotal factor associated with immune reconstitution after ART (75, 76). miR-16-5p has been shown to inhibit cell proliferation in different types of cancers by targeting *SMAD3* or cell cycle-related genes (77–81). We found that miR-16-5p could suppress Ca^2+^ influx in CD4^+^ T cells of people infected with HIV who had received ART. Ca^2+^ plays an essential part in controlling multiple key cellular processes, such as the development of T cells (82, 83). Therefore, our data suggest that miR-16-5p can inhibit T cell proliferation by regulating Ca^2+^ influx, resulting in the poor recovery of CD4^+^ T cell counts during ART. Other miRNAs in the panel, such as miR-138-5p, have been reported to be related to immune regulation in tumors and the inflammatory response (84–86). The expression of miR-323-3p was high in T cells producing inflammatory factors (e.g., interleukin (IL)-17 and IL-22) (87), which suggests it may have potential roles in regulating the immune response in HIV infection. Further studies to elucidate the regulatory functions of the four other miRNAs we identified could also shed more light on the molecular mechanisms of non-response to ART in people with HIV infection.

In conclusion, we established a novel, five-miRNA panel to predict ART-induced immune reconstitution in people with HIV infection. The combination of these five miRNAs showed high efficacy in predicting immune recovery after ART. Application of this biomarker panel before ART could help to identify INRs early. These miRNAs may also indicate new avenues for research into the molecular mechanisms underlying non-response, which could lead to better outcomes for INRs.

## Data Availability Statement

The raw data supporting the conclusions of this article will be made available by the authors, without undue reservation.

## Ethics Statement

The studies involving human participants were reviewed and approved by the ethical review committee from The First Hospital of China Medical University, Shenyang, China. Written informed consent to participate in the study was obtained from all individuals.

## Author Contributions

HS and ZN-Z conceived and designed the experiments. JN-L, JQ-L and YB-C carried out the experiments and analyzed the data. YY-R, YJ-F and YJ-J collected the samples and contributed reagents. HS, ZN-Z, JN-L and JQ-L prepared the manuscript. All authors contributed to the article and approved the submitted version.

## Funding

This work was supported by the National Natural Science Foundation of China [81871708], Mega-Projects of National Science Research for the 12^th^ Five-Year Plan [2015ZX10004801-002] and the Mega-Projects of National Science Research for the 13^th^ Five-Year Plan [2017ZX10201101].

## Conflict of Interest

YB-C is an employee of Beijing Quantobio Star Biotechnology Co., Ltd. (Beijing, China).

The remaining authors declare that the research was conducted in the absence of any commercial or financial relationships that could be construed as a potential conflict of interest.

## Publisher’s Note

All claims expressed in this article are solely those of the authors and do not necessarily represent those of their affiliated organizations, or those of the publisher, the editors and the reviewers. Any product that may be evaluated in this article, or claim that may be made by its manufacturer, is not guaranteed or endorsed by the publisher.

## Supplementary Material

Supplementary Figure 1 Relative expression of five plasma microRNAs (miRNAs) in the training set. Relative expression of five miRNAs in immune non-responders (INRs, n = 41) and immune responders (IRs, n = 85) in the training set.

Supplementary Figure 2 KEGG analysis by DIANA-miRPath v3.0. Heatmaps of signaling-pathway enrichment of five miRNAs **(A)** and miR-16-5p **(B)** using the KEGG database by Tarbase and microT-CDS.

Supplementary Figure 3 Efficacy of miR-16-5p overexpression in primary T cells. T cells from people with human immunodeficiency virus (HIV) infection (n = 7) after antiretroviral therapy (ART) were transfected with miR-16-5p mimic or control (10nM). The efficacy of miR-16-5p overexpression was confirmed by RT-qPCR. Data are presented as the mean ± SD.

Supplementary Figure 4 Overexpression of miR-16-5p does not affect T cell apoptosis. T cells from people with human immunodeficiency virus (HIV) infection (n = 9) after antiretroviral therapy (ART) were transfected with miR-16-5p mimic or control. Representative flow-cytometry data **(A)** and histogram **(B)** showing apoptosis of CD4^+^ T cells (top) and CD8^+^ T cells (bottom) measured using Annexin V and 7-Aminoactinomycin D.

Supplementary Figure 5 Matrix metalloproteinase-9 (MMP-9) decreased in the miR-16-5p overexpressed Jurkat cells. Relative quantitation of MMP-9 expression at protein level between miR-16-5p overexpressed (miR-16-5p mimic) and control Jurkat cells (n = 3). Label-free quantification (LFQ) intensity in different samples was centralized to obtain the relative quantitation value of protein.
