# Supplementary figures and images for: Plasma MicroRNA Signature Panel Predicts the Immune Response After Antiretroviral Therapy in HIV-Infected Patients
Source: Front Immunol. 2021 Nov 23;12:753044. doi: 10.3389/fimmu.2021.753044 (PMC8650117; doi:10.3389/fimmu.2021.753044)

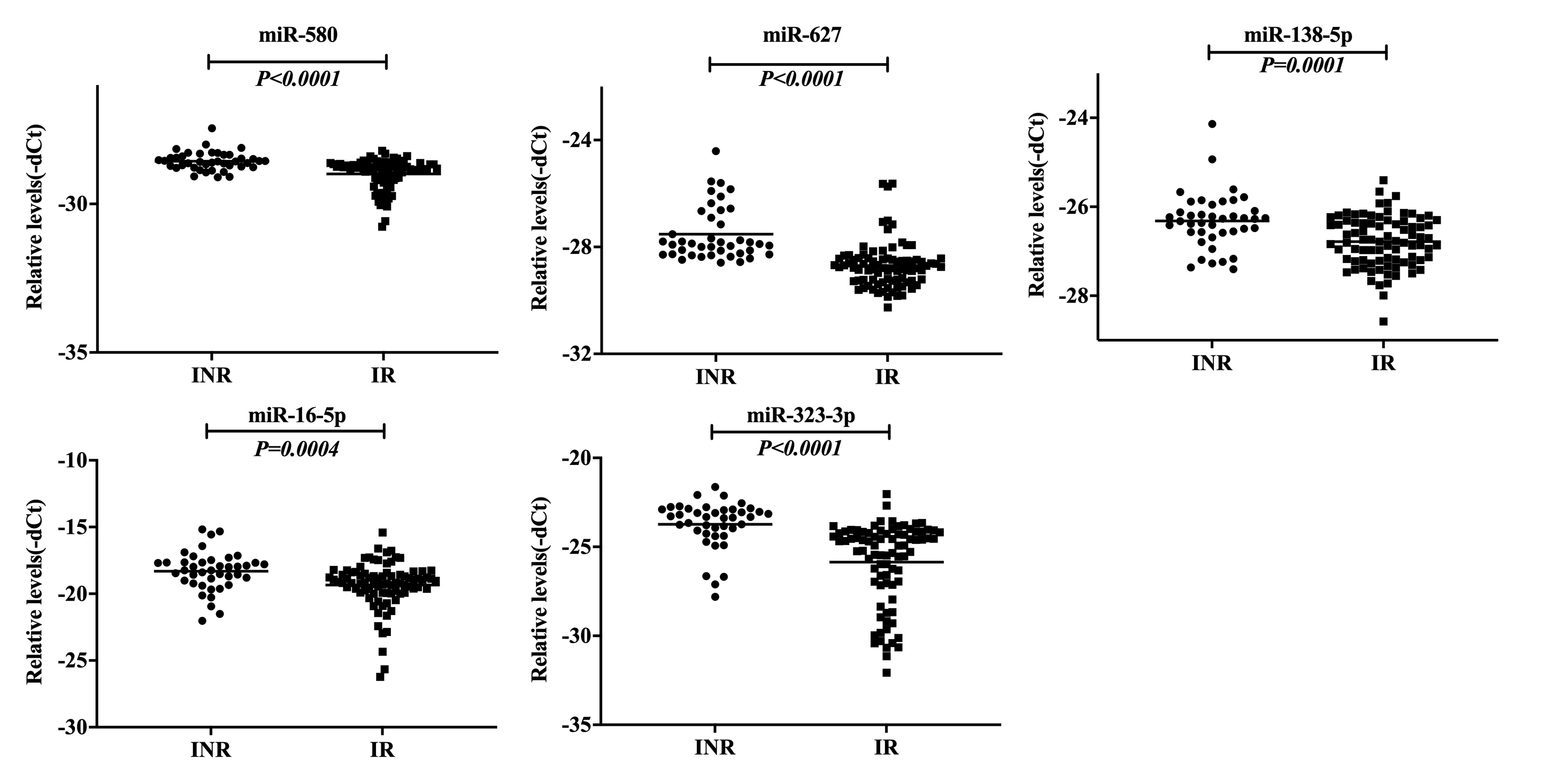

Supplement: Supplementary Figure 1 — Relative expression of five plasma microRNAs (miRNAs) in the training set. Relative expression of five miRNAs in immune non-responders (INRs, n = 41) and immune responders (IRs, n = 85) in the training set. [file Image_1.tiff]

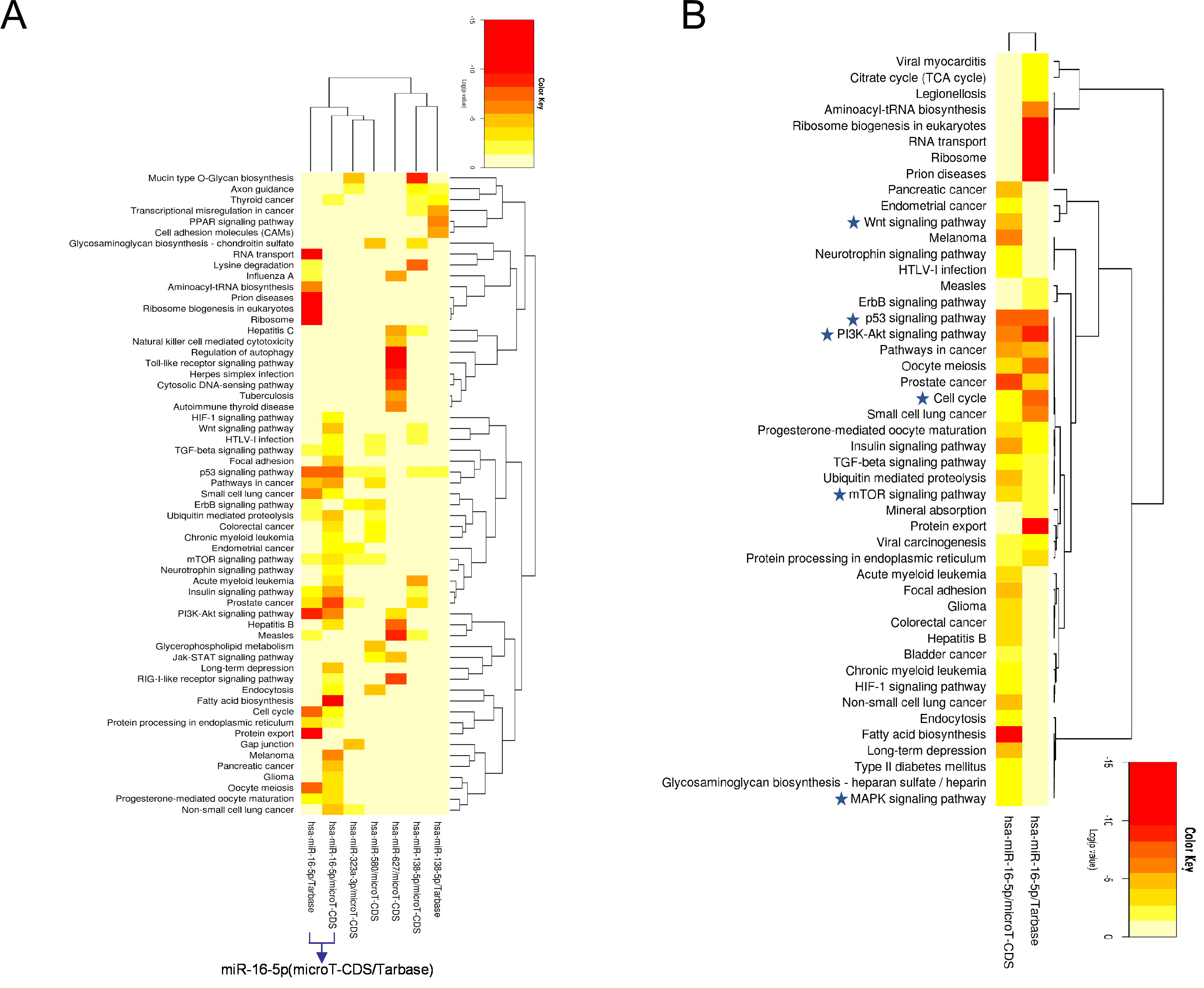

Supplement: Supplementary Figure 2 — KEGG analysis by DIANA-miRPath v3.0. Heatmaps of signaling-pathway enrichment of five miRNAs (A) and miR-16-5p (B) using the KEGG database by Tarbase and microT-CDS. [file Image_2.tif]

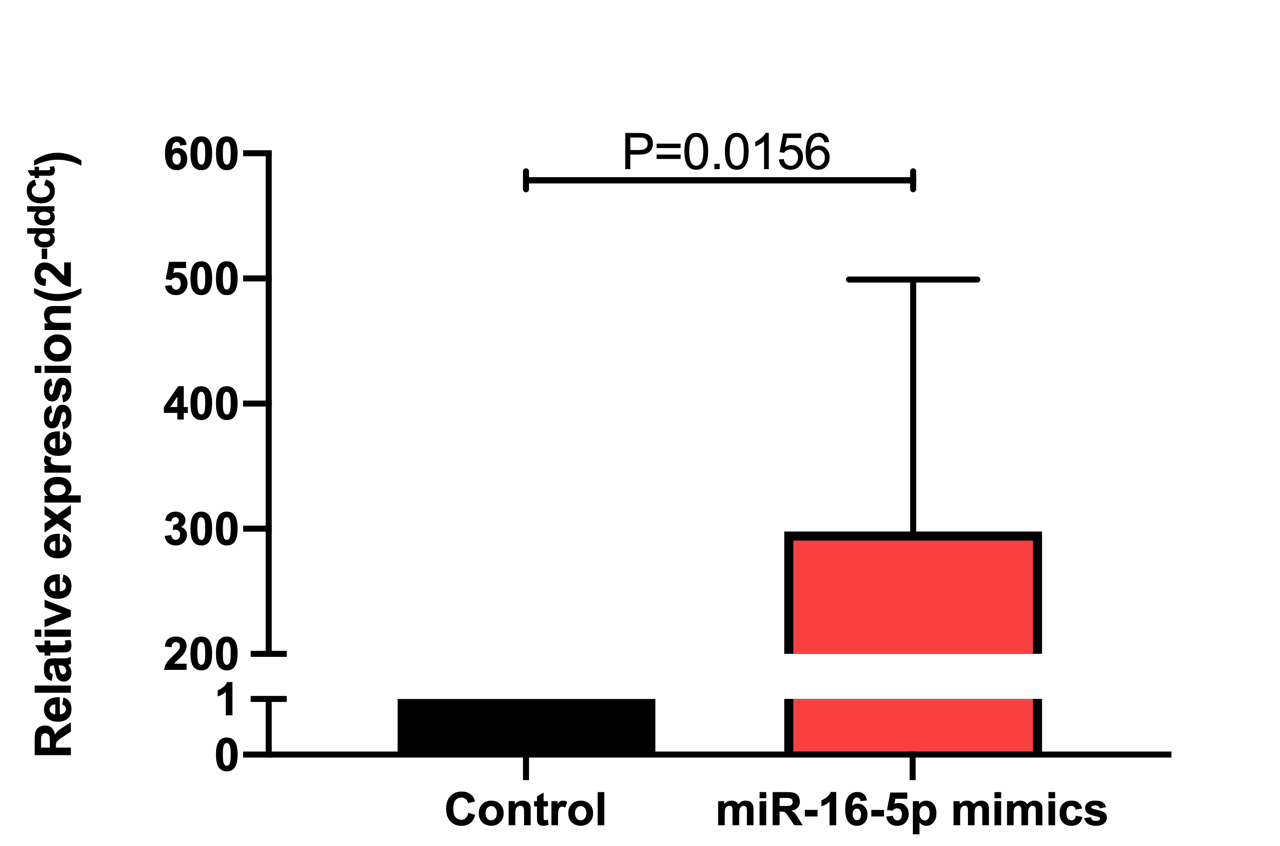

Supplement: Supplementary Figure 3 — Efficacy of miR-16-5p overexpression in primary T cells. T cells from people with human immunodeficiency virus (HIV) infection (n = 7) after antiretroviral therapy (ART) were transfected with miR-16-5p mimic or control (10nM). The efficacy of miR-16-5p overexpression was confirmed by RT-qPCR. Data are presented as the mean ± SD. [file Image_3.tiff]

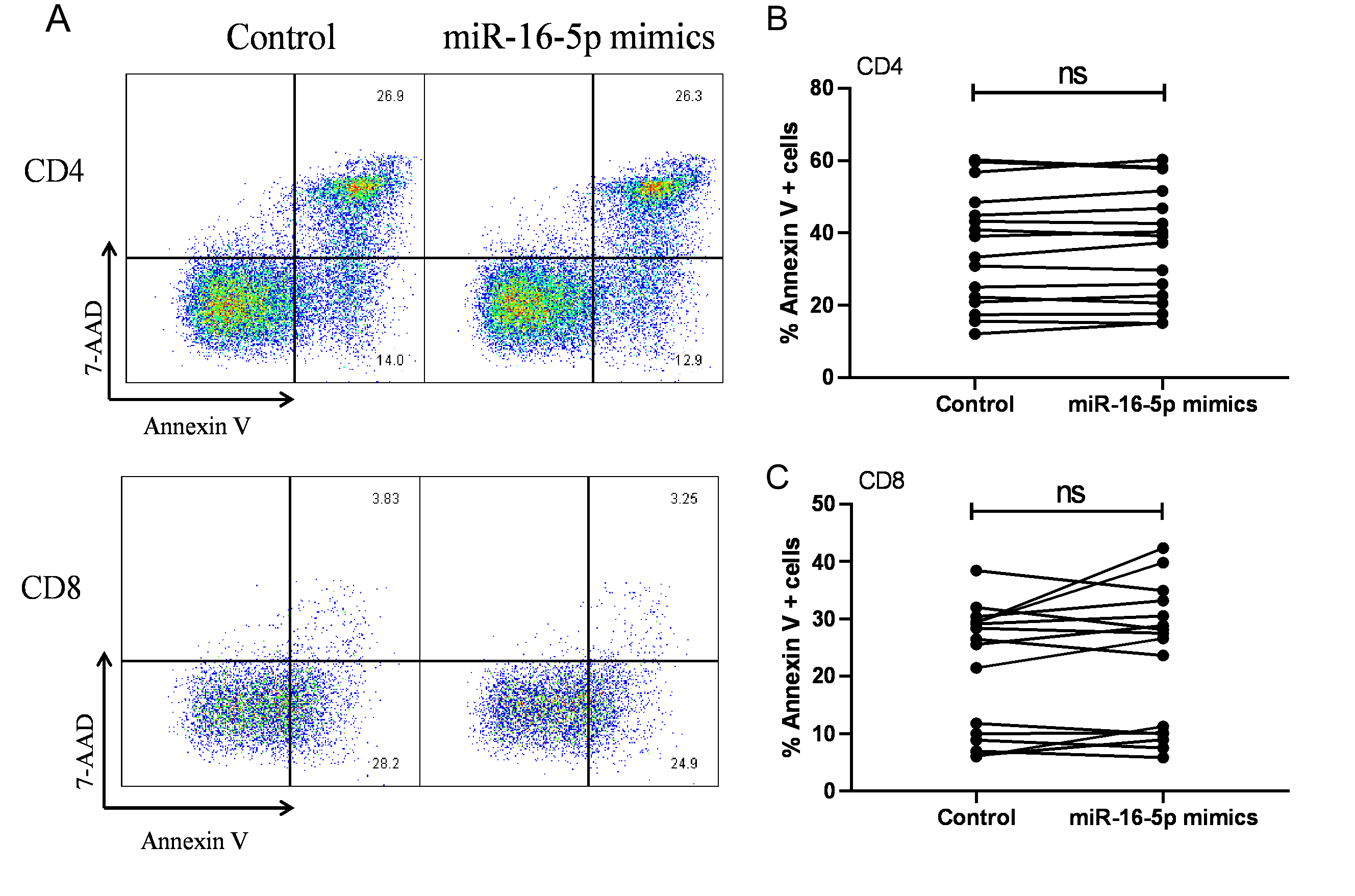

Supplement: Supplementary Figure 4 — Overexpression of miR-16-5p does not affect T cell apoptosis. T cells from people with human immunodeficiency virus (HIV) infection (n = 9) after antiretroviral therapy (ART) were transfected with miR-16-5p mimic or control. Representative flow-cytometry data (A) and histogram (B) showing apoptosis of CD4+ T cells (top) and CD8+ T cells (bottom) measured using Annexin V and 7-Aminoactinomycin D. [file Image_4.tif]

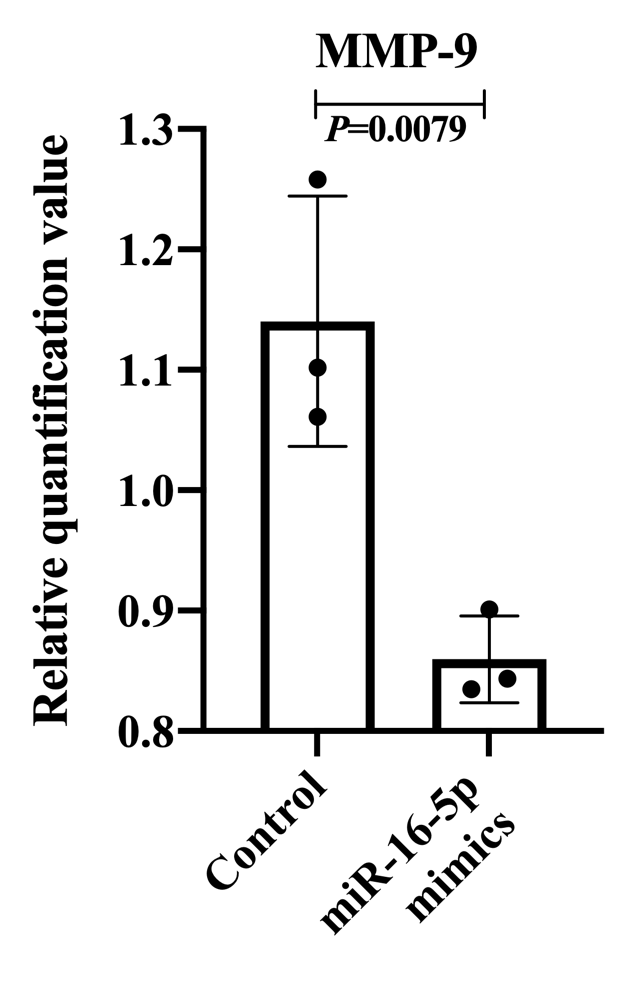

Supplement: Supplementary Figure 5 — Matrix metalloproteinase-9 (MMP-9) decreased in the miR-16-5p overexpressed Jurkat cells. Relative quantitation of MMP-9 expression at protein level between miR-16-5p overexpressed (miR-16-5p mimic) and control Jurkat cells (n = 3). Label-free quantification (LFQ) intensity in different samples was centralized to obtain the relative quantitation value of protein. [file Image_5.tiff]
