# Supplementary material for: Plasma MicroRNA Signature Panel Predicts the Immune Response After Antiretroviral Therapy in HIV-Infected Patients
Source: Front Immunol. 2021 Nov 23;12:753044. doi: 10.3389/fimmu.2021.753044 (PMC8650117; doi:10.3389/fimmu.2021.753044)
Supplement: Supplementary file 6 [file Table_1.docx]

|  | Sequence |
| --- | --- |
| EC |  |
| cel-miR-67-3p | 5’-UCACAACCUCCUAGAAAGAGUAGA-3’ |
| UPM |  |
| UPM-short | 5’-CTCACACGACTCACGACAC-3’ |
| UPM-long | 5’-CTCACACGACTCACGACACCAGTGGTATCAACGCACTC-3’ |
| miR-16-5p |  |
| mimics | 5’-UAGCAGCACGUAAAUAUUGGCGCCAAUAUUUACGUGCUGCUAUU-3’ |
| control |  |
| sense | 5’-UUCUCCGAACGUGUCACGUTT-3’ |
| anti-sense | 5’-ACGUGACACGUUCGGAGAATT-3’ |

**Supplemental table 1.** The sequences of used primers
